# ZnO NPs protect boar sperm in liquid storage through increasing the phosphorylation of PKAs

**DOI:** 10.1590/1984-3143-AR2024-0025

**Published:** 2025-04-18

**Authors:** Yuanyou Li, Meiling He, Haohan Ran, Jie Wu, Jing Lv, Guangyu Liu, Yifan Wang, Zhongliang Jiang

**Affiliations:** 1 Laboratory of Gamete Biology, College of Animal Science and Technology, Northwest A&F University, Yangling, Shaanxi, China; 2 Key laboratory of Bio-resources and Eco-environment of Ministry of Education, College of Life Sciences, Sichuan University, Chengdu, Sichuan, China

**Keywords:** boar sperm, liquid storage, ZnO NPs, ROS, protein phosphorylation

## Abstract

It remains a problem to efficiently improve the boar sperm quality of liquid storage due to reactive oxygen species (ROS) accumulation. To reduce the effects of ROS on boar sperm, in this study, 1 μg/mL zinc oxide nanoparticles (ZnO NPs) was added into the extender of boar semen during liquid storage at 4°C and 17°C for 7 days. The finding revealed that sperm motility, viability, plasma membrane integrity (PMI) and acrosome integrity significantly increased when compared with the control group (*P* ˂ 0.05) Additionally, ZnO NPs significantly increased the levels of adenosine triphosphate (ATP), mitochondrial membrane potential (MMP), and antioxidation abilities (*P* ˂ 0.05) in boar sperm. Moreover, ZnO NPs could protect boar sperm from oxidative stress (OS) by inhibiting ROS-induced decrease of phosphorylation of PKA substrates (P-PKAs). Together, the current results suggest that ZnO NPs could be used as a novel antioxidant agent for semen preservation, which is helpful in improving the application of assisted reproductive technology in domestic animals.

## Introduction

As a crucial component of the artificial insemination (AI) program, sperm storage is one of the effective methods for the preservation of boar genetic resources (Watson, 2000) and makes a substantial contribution to the advancement of animal industry and medical research (Li et al., 2020). Although sperm cryopreservation can prolong the survival of boar sperm in vitro, only 1% of the AI with frozen-thawed boar sperm is successful worldwide due to freezing injury (Didion et al., 2013). Thus, to avoid boar sperm from being subjected to freezing injury and to prolong sperm survival time in vitro, many researchers are committed to studying liquid storage of boar sperm at 4 ~ 17°C.

**Figure 1:**
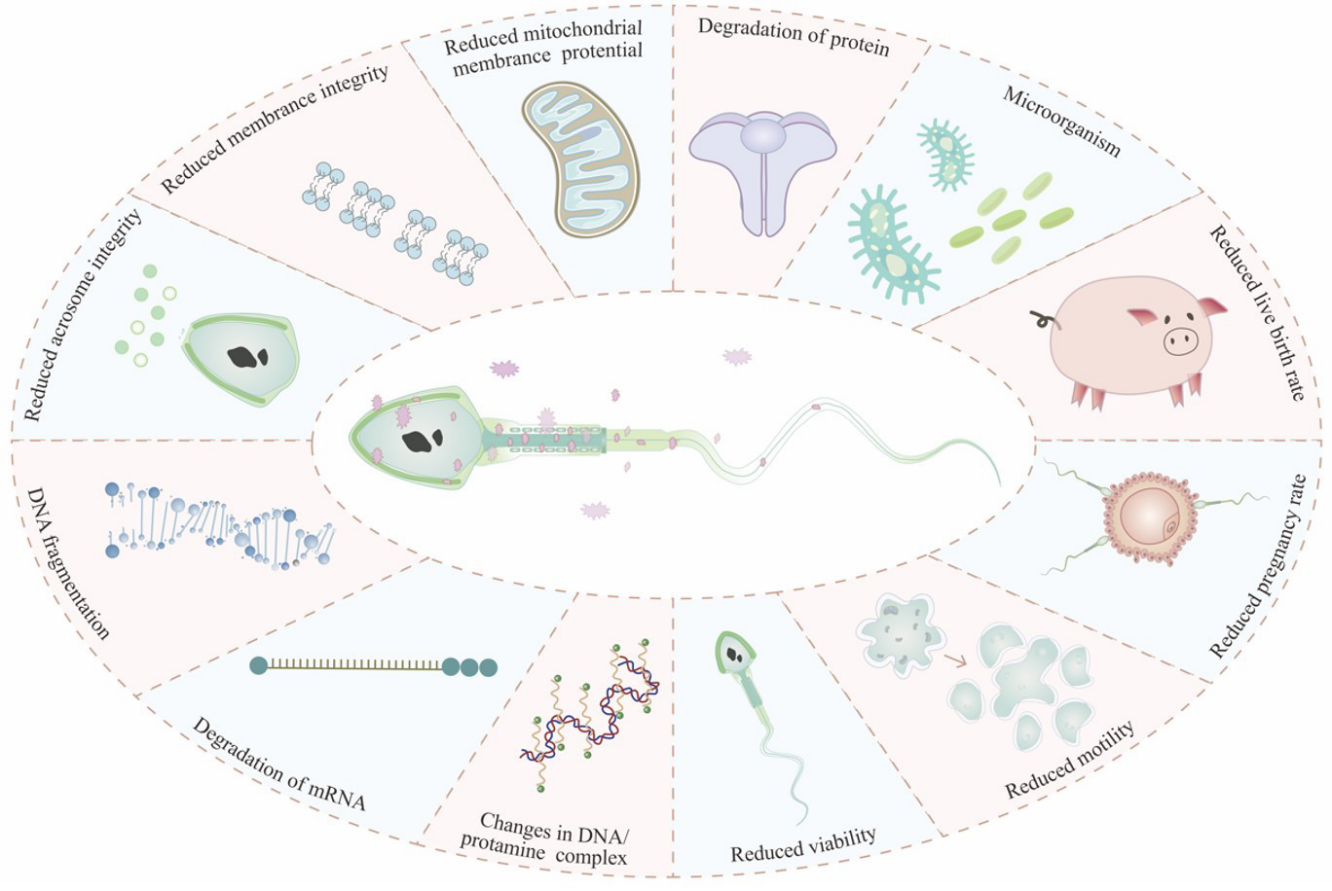
The consequences of damage on sperm in liquid storage.

In sperm, besides ATP, reactive oxygen species (ROS) are generated as by-products of mitochondrial oxidative phosphorylation, which causes oxidative damage in mitochondria during liquid storage (Piomboni et al., 2012). High levels of ROS can cause infertility not only by lipid peroxidation or DNA damage but also by inactivation of enzymes and cause oxidative stress (OS) (Barati et al., 2020; Gadani et al., 2017). Due to a low cholesterol/phospholipid ratio in the plasma membrane, ineffective free radical scavenging system, and the lack of cytoplasm in the boar sperm, boar sperm are particularly vulnerable to OS (Aitken et al., 1989; White, 1993). The liquid storage-related damage on boar sperm is shown in Figure 1. Therefore, regulation of the generation of excessive ROS to eliminate the ROS-induced damage to the structural integrity of the sperm membrane and acrosome, and maintenance of the stability of the genetic material during liquid storage (Zhu et al., 2019a) are serious challenges. Despite significant attempts to find exogenous antioxidants to prevent oxidative damage, which suggested that adding antioxidant agents to extender can protect sperm from OS and improve sperm quality metrics (Fu et al., 2018; Gadani et al., 2017; Li et al., 2019, 2022), there is still no consolidated conclusion.

Nanoparticles have lengths ranging from 1 to 100 nanometers in two or three dimensions with highly customizable physical and optical qualities, and the ability to bind ligands from an ever-expanding library (Barkhordari et al., 2013; Yohan & Chithrani, 2014). Zinc oxide nanoparticles (ZnO NPs), one of the most prevalent nanomaterials, have been widely used in various industries due to its ultraviolet-protective, outstanding antibacterial, and antimicrobial capabilities (Choi & Choy, 2014; Jiang et al., 2018). Notably, ZnO NPs is known to be a putative antioxidant with a powerful antioxidant ability, which can prevent tissue dysfunction induced by OS (Azarin et al., 2022; Bashandy et al., 2018; Erfani Majd et al., 2021; Faizan et al., 2021; Gharaei et al., 2020; Liu et al., 2022; Mahesh et al., 2022). Additionally, many beneficial effects of ZnO NPs have been observed even at extremely low doses (Abdelbaky et al., 2022; Farhana et al., 2022; Kalaimurugan et al., 2022). Despite substantial experimental results demonstrating the beneficial effect of ZnO NPs on biological systems in vivo and in vitro, few attempts have been made to comprehend the molecular mechanism of its antioxidant function. The effects of ZnO NPs on the quality of liquid-storage sperm, particularly, are yet to be investigated.

In the present study, we explore the effects of ZnO NPs on the quality of liquid-storage boar sperm, and whether ZnO NPs could exert significant antioxidant effects on boar sperm at 4°C and 17°C. Herein, we systematically assessed the protective role of ZnO NPs against oxidative damage. Our results demonstrated the good antioxidative capacity of ZnO NPs and its great potential used as a semen extender additive. Simultaneously, this study provides a theoretical basis for broadening the use of nanoparticles as antioxidants in the field of animal reproduction.

## Methods

### Experimental design

Experiment 1 was designed to investigate whether ZnO NPs has beneficial effects on boar sperm functionality during storage at 4°C and 17°C. Each ejaculate was stored in the basal medium (Table 1) and ZnO NPs suspension was added into the samples to make a final concentration of 0.1, 1, 10 μg/mL at 4°C and 17°C. In this experiment, we analyzed sperm motility, viability, acrosome integrity, plasma membrane integrity (PMI), and abnormality rate after 7 days of storage.

**Table 1:** The basal extender for liquid storage of boar sperm.

**Component**	**Content g/100 mL**
Glucose	4.000
Trisodium citrate	0.780
EDTA	0.150
Sodium bicarbonate	0.080
Citric acid	0.023
Potassium chloride	0.070
Gentamicin sulfate	0.030

Experiment 2 sought to confirm the antioxidant capabilities of ZnO NPs on liquid-storage boar sperm. ZnO NPs suspension was added to a final 1 μg/mL concentration in the basal medium. After 7 days of liquid storage at 4°C and 17°C, we analyzed sperm mitochondrial membrane potential (MMP), ATP level, ROS level, Malondialdehyde (MDA) level, total antioxidant capacity (T-AOC), superoxide dismutase (SOD), glutathione peroxidase (GSH-Px), catalase (CAT) activities, and protein phosphorylation levels in this experiment.

Experiment 3 was devised to elucidate whether ZnO NPs protected boar sperm from suppressing ROS-induced protein dephosphorylation. 100 μM concentrations of hydrogen peroxide (H_2_O_2_) was added to the basal medium that contained 1 μg/mL ZnO NPs. The expression of protein tyrosine phosphorylation (PTP), P-PKAs, and ROS levels were detected by western blotting and fluorescent microscope.

### Chemicals and preparation of ZnO NPs suspension

**Figure 2:**
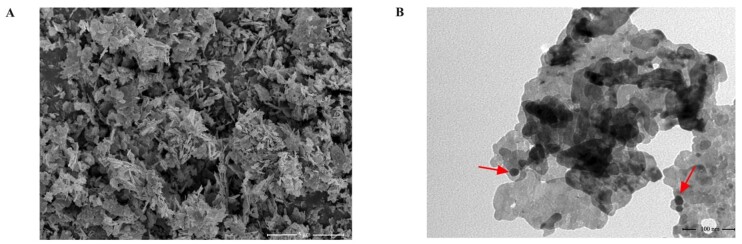
The SEM (A) and TEM (B) images of the ZnO NPs used in the experiment. The red arrow indicates ZnO NPs.

Unless otherwise mentioned, all the chemical products were obtained from Sigma-Aldrich (St. Louis, MO, USA). ZnO NPs with a purity of > 99.98% were obtained from Macklin Biochemical Technology Co., Ltd. (Shanghai, China). Transmission electron microscopy (TEM, HT7800, acceleration voltage = 80 KV) was applied to characterize the size of nanoparticles. A scanning electron microscope (SEM, FEI) was applied to characterize the morphology of nanoparticles. The particles exhibited configurations characterized by flower-like shapes, encompassing a dimensional range of 28.65 ± 3.47 nm (Figure 2). ZnO NPs were meticulously suspended within PBS, culminating in a concentration of 10 mg/mL. PBS was meticulously employed as a suspension agent, serving the dual purpose of achieving homogenous nanoparticle dispersion and enhancing ease of handling. The resultant suspension underwent thorough ultrasonic dispersion within an ice bath, a process meticulously executed for a duration of 30 minutes prior to each application.

### Animals

All the animal experiments strictly adhered to the standards of the institutional guidelines for ethics in animal experimentation (Rule number 86/609/EEC-24/11/86). All the experimental procedures were approved by the Animal Ethics Committee of Northwest Agriculture and Forestry University (approval No. XN2023-0615). Eight healthy and sexually mature Duroc boars, with ages ranging from 1.5 to 2 years and proven fertility, were used in this study. The Duroc boars were housed individually, maintained under natural daylight, and provided with free access to food and water.

### Semen collection and processing

Semen samples were collected from 8 Duroc boars with the gloved hand technique, the fresh semen was placed in a 37°C bath and delivered to the laboratory within 30 min for evaluation using the computer-assisted semen analysis (CASA) system (Integrated Semen Analysis System; Hview, Fuzhou, China). Only ejaculates with more than 70% motile sperm and more than 80% morphologically normal sperm were used in this study. Each ejaculate was diluted in the basal extender (Table 1). All the samples reached a final concentration of 1 × 10^8^ cells/mL and were stored for 7 days at 4°C and 17°C, respectively.

### Sperm quality assessment

#### Sperm motility, viability, and abnormality rate

After 7 days of liquid storage, 1 mL of each sample was taken from each bottle and incubated at 37°C for 30 min before evaluation. After incubation, 7 μL of every 1 mL semen sample was placed on pre-warmed disposable counting chamber slides (Leja, Nieuw Vennep, the Netherlands). CASA system was used to measure sperm viability, motility, and abnormality rate. The analysis was performed with images from several fields containing at least 300 sperms per sample, and the sample analysis was based on the examination of at least 9 images.

#### Sperm PMI

Sperm PMI was evaluated using a LIVE/DEAD Sperm Viability Kit (L7011; Thermo Fisher Scientific, USA), which contains a membrane-permeant nucleic acid stain developed at Molecular Probes (SYBR® 14 dye), and the conventional dead cell stain, propidium iodide (PI). Briefly, According to the previous studies (Ren et al., 2019; Zhang et al., 2023). Added 5 μL of diluted SYBR 14 dye to a 1 mL sample of diluted semen, resulting in a final SYBR 14 concentration of 100 nM. Incubate for 10 minutes at 36°C. Then, added 5 μL of PI to the 1 mL sample of diluted semen, resulting in the final PI concentration will be 12 μM. Incubate for 10 minutes at 36°C and the sperm plasma membrane was visualized with the fluorescent microscope (Leica DMI8, Germany). The fluorescence emission maxima of SYBR 14 and PI are 516 nm and 617 nm, respectively. Live sperm cells with intact cell membranes fluoresced bright green, while sperms with damaged cell membranes fluoresced red. Each slide contains at least 200 sperm from three different fields of vision. Take at least three measurements per time. The analysis was done in triplicate (n=3).

#### Sperm acrosome integrity

Fluorescein isothiocyanate-peanut agglutinin staining (FITC-PNA) (L7381, Sigma-Aldrich, USA) and 4',6-diamidino-2-phenylindole (DAPI) (C1005, Beyotime Institute of Biotechnology, Shanghai, China) were used to detect the integrity of boar sperm acrosome. 100 μL sperm samples were washed with PBS, spreaded on a glass slide with poly-D-lysine, dried naturally, and fixed in anhydrous methanol for 10 min. Then, a volume of 30 μL DAPI (4.8 μmol/L in PBS) staining solution was evenly spreaded onto each slide and air dry. Add equal volume of FITC-PNA (30 μL, 100 μg/mL in PBS ) staining solution was evenly spread onto each slide and incubated in darkness at 37°C for 30 min. After incubation, gently rinse the slide with PBS and air dry. Finally, acrosome integrity was observed using a fluorescence microscope (Leica DMI8, Germany). Sperm with an intensively bright green fluorescence of the acrosomal cap were deemed to have an intact outer acrosomal membrane; spermatozoa with a disrupted fluorescence of the acrosomal cap or no fluorescence of the outer acrosomal membrane were deemed to have a damaged acrosome membrane. Each slide contained at least 200 sperm from three different fields of view. Perform at least three measurements each time, and the results are calculated as the average of the three replicates.

#### Sperm MMP

The MMP (Δψm) was measured by JC-1 Mitochondrial Membrane Potential Detection Kit (Beyotime Institute of Biotechnology, Shanghai, China). The fluorescent carbocyanine dye, JC-1, labeled mitochondria with high membrane potential red and mitochondria with low membrane potential green. The ratio of this green/red fluorescence is independent of mitochondrial shape, density, or size, but depends only on the membrane potential. Briefly, 100 μL sperm samples were centrifuged at 106 × g for 5 min at 4°C, and the supernatant was removed and washed three times with PBS for 5 min each. Sperm were stained with 1 x JC-1 (10 μg/mL) and incubated at 37°C for 30 min under light-protected conditions. Then, the samples were washed three times with PBS. The samples were observed using a fluorescence microscope (Leica DMI8, Germany) (Zhu et al., 2019c) at an excitation wavelength (515 nm or 585 nm), each slide contained at least 200 sperm from three different fields of view. The MMP was calculated as the fluorescence ratio of red (aggregates) to green (monomers). The analysis was done in triplicate (n=3).

#### Sperm ATP content

The level of ATP was determined using an ATP assay kit (A095, Nanjing Jiancheng, China), according to the manufacturer’s protocols. 1 mL aliquots containing 5×10^7^ sperm samples were centrifuged and resuspended in ATP assay lysate to release intracellular ATP on ice. Sperm counts were performed for every sample to normalize ATP content to sperm number. Samples were centrifuged at 1000 × g for 5 min at 4°C. The ATP standard solution (5 mmol/L) was diluted to concentrations of 10 nmol/L to 10 µmol/L in succession by ATP assay lysate. Either supernatants or standards (lysate at the same volume as the blank) were added to luciferin/luciferase reagent in opaque 96-wells, and the fluorescence intensity of samples was detected by a multi-detection microplate reader (Synergy H1, BioTek, USA) at an excitation wavelength (636 nm). At least three technical replicates were evaluated for each sample.

#### Sperm intracellular ROS level

The ROS level in sperm samples was evaluated using the probe DCFH-DA (S0101S, Beyotime Institute of Biotechnology, Shanghai, China), according to the manufacturer’s protocols. Briefly, 100 μL sperm sample was treated with DCFH-DA, resulting in a final DCFH-DA concentration of 100 nM, and incubated in darkness at 37°C for 20 min. Then, the mixture was centrifuged to remove the supernatant. The fluorescence intensity of samples was immediately measured using the fluorescent microscope (Leica DMI8, Germany) at an excitation wavelength (488 nm), each slide contained at least 200 sperm from three different fields of view. At least three technical replicates were evaluated for each sample.

#### Sperm intracellular MDA level

MDA concentration as indices of lipid peroxidation in semen samples was measured by the Thiobarbituric acid (TBA) reaction using MDA assay kits (A003, Nanjing Jiancheng, China). 250 μL sperm samples were centrifuged to discard the supernatant. Added 1 mL of basic extender, 0.25 mL ferrous sulfate (0.2 mM), and 0.25 mL ascorbic acid (1 mM), and incubated at 37°C for 1 h after which 1 mL trichloroacetic acid (15%) and 1 mL thiobarbituric acid (0.375%) were added. Then, boil the mixture in water for 10 min. Thereafter, the mixture was cooled to room temperature to stop the reaction. Then, the mixture was centrifuged, and the supernatant was removed and washed three times with PBS for 5 min each. The fluorescence intensity of samples was detected by a multi-detection microplate reader (Synergy H1, BioTek, USA) at Excitation/Emission = 532 nm. At least three technical replicates were evaluated for each sample.

#### Sperm intracellular SOD, GSH-Px, CAT activities

The activities of SOD, GSH-Px, and CAT in sperm were determined using, the total SOD assay kit (Nanjing Jiancheng, China), Glutathione Peroxidase (GSH-PX) assay kit (Nanjing Jiancheng, China), CAT assay kit (Nanjing Jiancheng, China), according to Zhu et al. (2019b). The sperm samples were rinsed three times with PBS (106 × g for 5 min at 4°C) and resuspended, then lysed ultrasonically (20 kHz, 750 W, operating at 40%, on 3 s, off 5 s, 5 cycles) on ice and centrifuged at 17949 × g for 10 min at 4°C. The total protein concentration was practiced as the BCA protein assay kit (TaKaRa, Japan). The supernatants were used to analyze the GSH-Px, SOD, and CAT activities according to the manufacturer’s instructions. SOD, GSH-Px, and CAT activities in sperm were represented as mU/mg or U/mg protein (prot.). All experiments were carried out in quadruplicate (n = 4).

### Western blotting

Sperm samples were first centrifuged at 1,300 rpm at room temperature for 5 min, then washed with PBS and resuspended with RIPA buffer containing 1% phenylmethyl sulfonylfluoride (PMSF) and phosphatase inhibitor and 1% protease inhibitor cocktail (EDTA free, 100×; MedChemExpress, China) for 30 min at 4°C. Given that the sperm membrane is relatively unbreakable, the samples were lysed by ultrasonication (20 KHz, 750 W, operating at 30% power, six cycles for 5 s on and 5 s off). After 30 min of lysis at 4°C, the samples were centrifuged at 19357 × g for 15 min at 4°C and the supernatant was transferred to a new centrifuge tube. A portion of the supernatant was used to analyze the concentration of total protein by spectrophotometry (NanoDrop 2000, Thermo Scientific, USA). The rest was mixed with 5 × SDS loading buffer and boiled at 95°C for 5 min.

The 30 μg of the extracted sperm protein was added to each lane of a 10% polyacrylamide gradient gel, then transferred onto PVDF membranes (Merck Millipore, Germany) at 15 V for 40 min. The membranes were blocked with QuickBlock™ Blocking Buffer (Beyotime Institute of Biotechnology, Shanghai, China) at room temperature for 1 h. After blocking, the membranes were immunoblotted with an anti-P-PKA antibody (Cell Signaling Technology, 1:1000, Cat# 9624, clone 100G7E), an anti-phosphotyrosine antibody (Millipore, 1:2000, Cat# 05-321, clone 4G10), or β-tubulin (ABclonal technology, 1:5000, Cat#AC021, China) followed by overnight incubation at 4°C respectively. The membranes were washed twice with TBST and then incubated with the anti-rabbit HRP-conjugated IgG goat (Proteintech Group, 1:10,000, RGAR001, China) for 1 h at room temperature. An enhanced chemiluminescence ECL-plus kit (GE Healthcare Worldwide, USA) was used to develop the signals, which were detected using a ChemiScope 3300 mini-integrated chemiluminescence imaging system (CLINX, China). For all the experiments, the molecular weights of the sperm proteins are indicated in kDa.

### Statistical analysis

All data are indicated as the mean ± standard error of the mean (SEM). The significance of differences in means was determined by a one-way analysis of variance (ANOVA) for multigroup comparisons. The significance of differences in means was determined by a t-test for two comparisons. Each experiment was set to be repeated at least 3 times. All the statistical analyses were performed using GraphPad 9.0 software. *P* value < 0.05 was considered statistically significant (*P < 0.05, **P < 0.01).

## Results

### Effects of ZnO NPs on the quality of boar sperm in liquid storage

**Figure 3:**
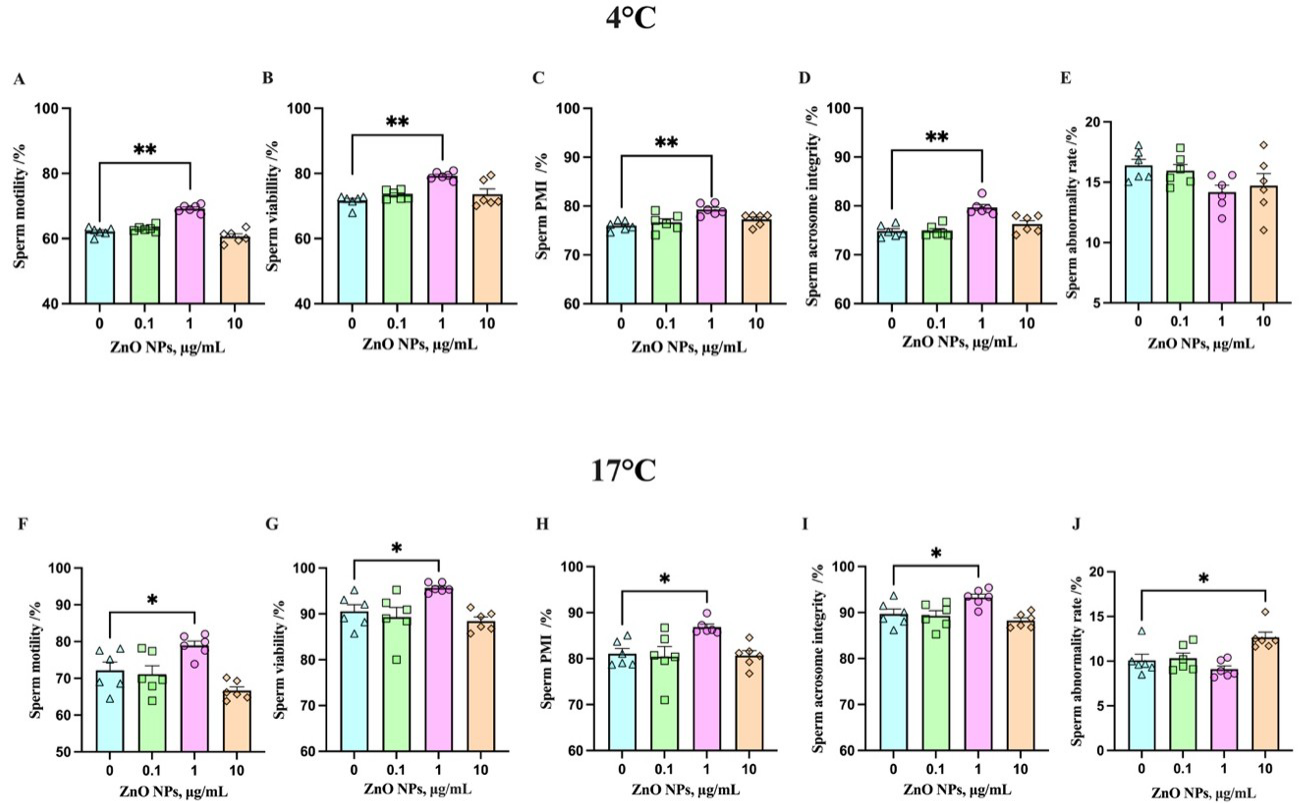
Effects of ZnO NPs on the quality of sperm after storage for 7 days at 4°C. The sperm motility (A), viability (B), plasma membrane integrity (PMI)(C), acrosome integrity (D), and abnormality rate (E) at 4°C are measured. The sperm motility (F), viability (G), PMI (H), acrosome integrity (I), and abnormality rate (J) at 17°C are shown. The data are expressed as mean ± SEM. (*n* = 6, * *P* < 0.05, ** *P* < 0.01).

To investigate whether ZnO NPs has beneficial effects on boar sperm quality in storage at 4°C and 17°C. Each ejaculate was diluented with basal extender and ZnO NPs suspension was added into the samples to make a final concentration of 0.1, 1, 10 μg/mL at 4°C and 17°C. After storage for 7 days, we investigated the effects of ZnO NPs on boar sperm quality in liquid storage (Figure 3). The results showed that 1 μg/mL ZnO NPs significantly increased the motility, viability, PMI, and acrosome integrity of boar sperm which keep in storage at 4°C (Figure 3A-D) and 17°C (Figure 3F-I). ZnO NPs did not change the abnormality rate of boar sperm preserved at 4°C (Figure 3E), while the higher concentration of 10 μg/mL ZnO NPs significantly increased the abnormality rate of boar sperm at 17°C (Figure 3J). Taken together, 1 μg/mL ZnO NPs had a better protective effect on liquid-storage sperm quality. Consistently, the concentration of 1 μg/mL ZnO NPs was selected for the next experiments in this study.

### Effects of ZnO NPs on boar sperm mitochondria in liquid storage

**Figure 4:**
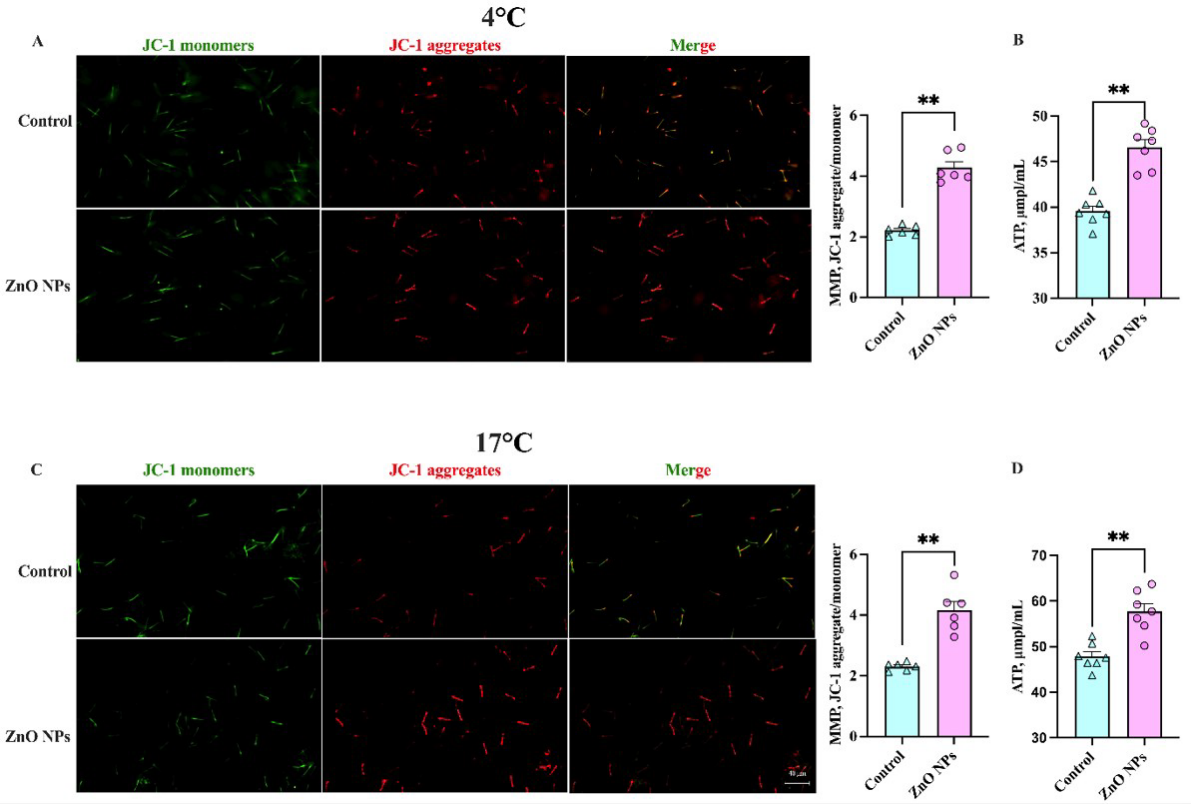
Effects of 1 μg/mL ZnO NPs addition on sperm mitochondrial membrane potential (MMP), and cellular ATP content after liquid storage at 4°C and 17°C for 7 days. (*n* = 6, ** *P* < 0.01). An Effect of adding 1 μg/mL ZnO NPs in sperm extender on the boar sperm MMP after 7 days of liquid storage at 4°C. B Effect of adding 1 μg/mL ZnO NPs in sperm extender on the boar sperm ATP after 7 days of liquid storage at 4°C. C Effect of adding 1 μg/mL ZnO NPs in sperm extender on the boar sperm MMP after 7 days of liquid storage at 17°C. D Effect of adding 1 μg/mL ZnO NPs in sperm extender on the boar sperm ATP after 7 days of liquid storage at 17°C.

To verify the potential protective effects of ZnO NPs against oxidative damage during liquid storage, we analyzed the effects of ZnO NPs on the mitochondria of boar sperm in liquid storage. JC-1 marker was used to stain the sperm preserved at 4°C (Figure 4A) and 17°C (Figure 4C), and the results showed that ZnO NPs significantly increased the sperm MMP at both 4°C (Figure 4B) and 17°C (Figure 4E), respectively. After 7 days of liquid storage at 4°C and 17°C, the ZnO NPs-treated group had higher ATP than the control group (*P* < 0.01) (Figure 4C, F). Thus, these results demonstrated that ZnO NPs could maintain sperm MMP, and ATP content during liquid storage at 4°C and 17°C in vitro.

### Effects of ZnO NPs on boar sperm antioxidant capabilities in liquid storage

**Figure 5:**
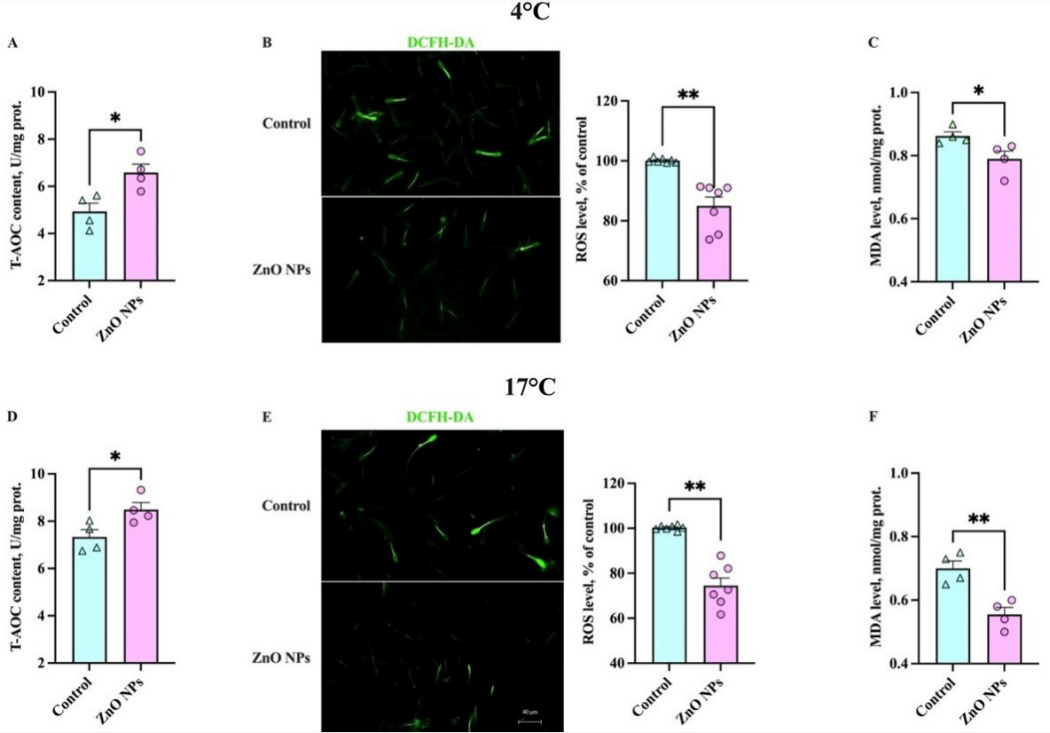
Effects of 1 μg/mL ZnO NPs on reactive oxygen species (ROS), total antioxidant capacity (T-AOC), and Malondialdehyde (MDA) levels of sperm after storage for 7 days at 4°C and 17°C. The sperm T-AOC (A), ROS (B), and MDA levels (C) at 4°C are measured. The sperm T-AOC (D), ROS (E), and MDA levels (F) at 17°C are shown. (*n* > 3, * *P* < 0.05, ** *P* < 0.01).

**Figure 6:**
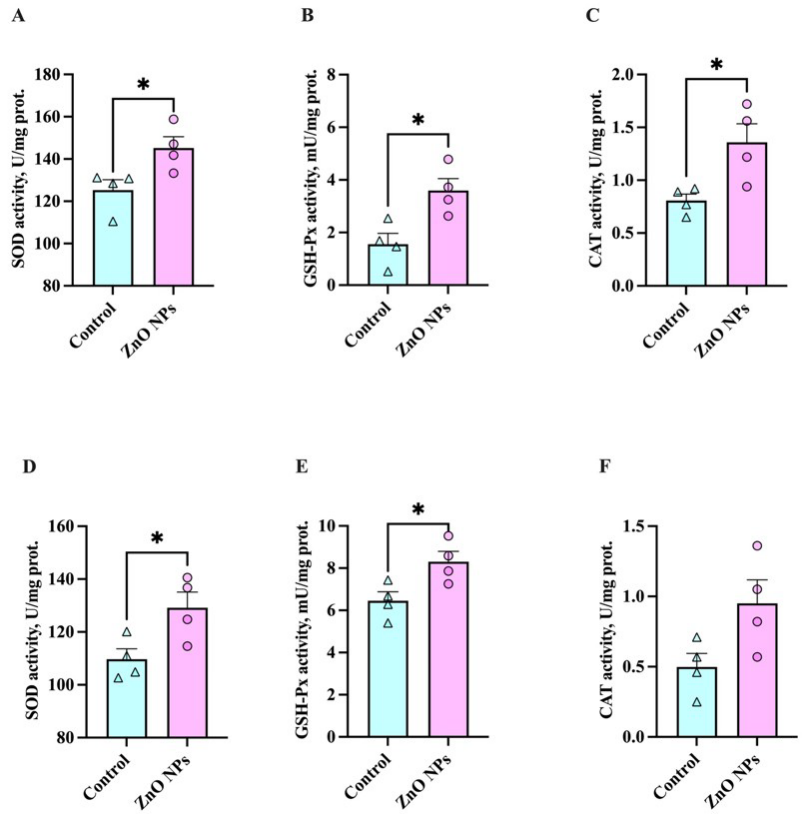
Effects of 1 μg/mL ZnO NPs on SOD, GSH-Px, and CAT activities of sperm after storage for 7 days at 4°C and 17°C. The sperm superoxide dismutase (SOD)(A), glutathione peroxidase (GSH-Px)(B), and catalase (CAT)(C) activities at 4°C are measured. The sperm SOD (D), GSH-Px (E), and CAT (F) activities at 17°C are shown. (*n* > 3, * *P* < 0.05).

To verify the potential antioxidant effects of ZnO NPs in liquid storage at 4°C and 17°C, we analyzed T-AOC, ROS, MDA levels (Figure 5), and antioxidant enzyme activities (Figure 6) of sperm samples. After 7 days of liquid storage at 4°C and 17°C, the increase in the level of T-AOC (Figure 5A, D) and the decrease in the level of ROS (Figure 5B, E) were observed in sperm incubated with ZnO NPs, respectively (*P* < 0.05). Similar results of MDA levels were found in the sperm in liquid storage (*P* < 0.05) (Figure 5C, F), indicating ZnO NPs could suppress sperm membrane peroxidation reaction. This result was in agreement with the results about sperm PMI. An ineffective antioxidant system is one reason for the vulnerability to the OS of boar sperm in liquid storage. Moreover, the activities of antioxidant enzymes in sperm were obviously increased in the samples of ZnO NPs addition in liquid storage at both 4°C (Figure 6A-C) and 17°C (Figure 6D, E), respectively. Consistently, these results demonstrated that ZnO NPs exhibited outstanding antioxidant capabilities during liquid storage.

### Effects of ZnO NPs on boar sperm protein phosphorylation in liquid storage

**Figure 7:**
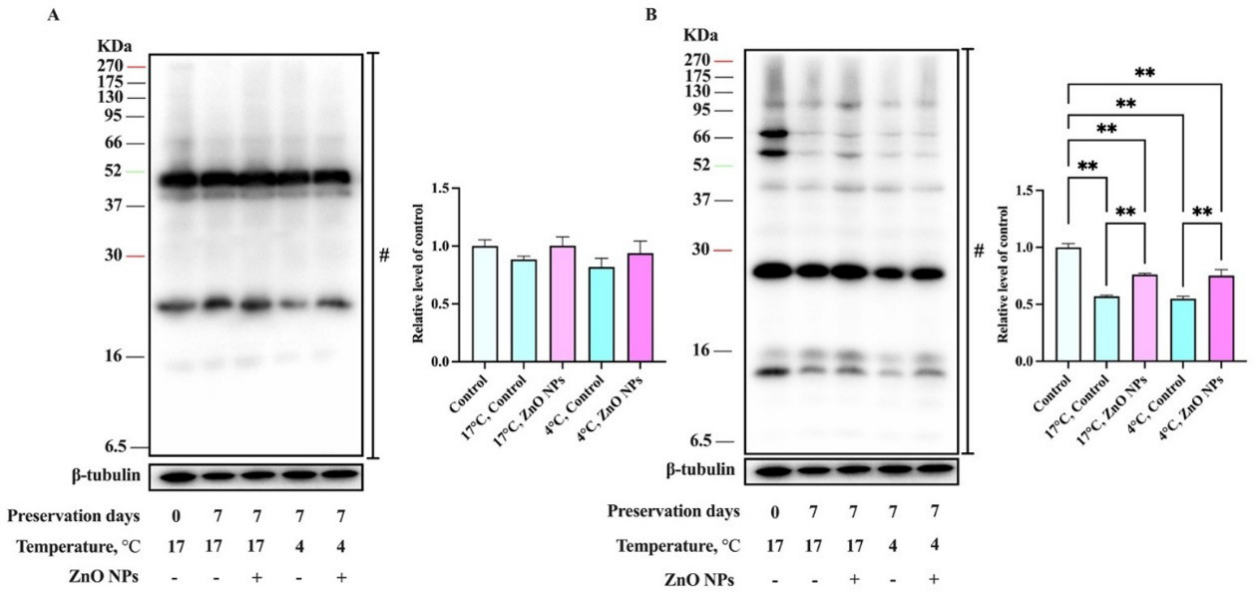
Effects of 1 μg/mL ZnO NPs on sperm PTP (A) and P-PKAs (B) levels during storage at 4°C and 17°C. A Western blot analysis was performed using an anti-phosphotyrosine antibody. B Western blot analysis was performed using an anti-phospho-PKA substrate antibody. The bands used for histogram quantification are labeled #. β-Tubulin was used as an internal control. The experiment was performed three times; the presented image is representative and repeatable. (n = 3, ** *P* < 0.01).

To understand the molecular mechanism of ZnO NPs on boar sperm survival, the protein tyrosine phosphorylation (PTP) and phosphorylation of PKA substrates (P-PKAs) were measured in this experiment. After 7 days of liquid storage, the level of PTP was no significantly different but P-PKAs was significantly decreased compared to the fresh sperm (Figure 7). Moreover, the results showed that ZnO NPs could noticeably increase the level of P-PKAs compared to that in the control group(*P* < 0.01) (Figure 7B). However, the level of P-PKAs after 7 days of storage was significantly lower than that of fresh sperm in both the treatment and control groups (*P* < 0.01) (Figure 7B). This outcome was consistent with sperm motility.

### ZnO NPs alleviate ROS-Induced Sperm P-PKAs depletion

**Figure 8:**
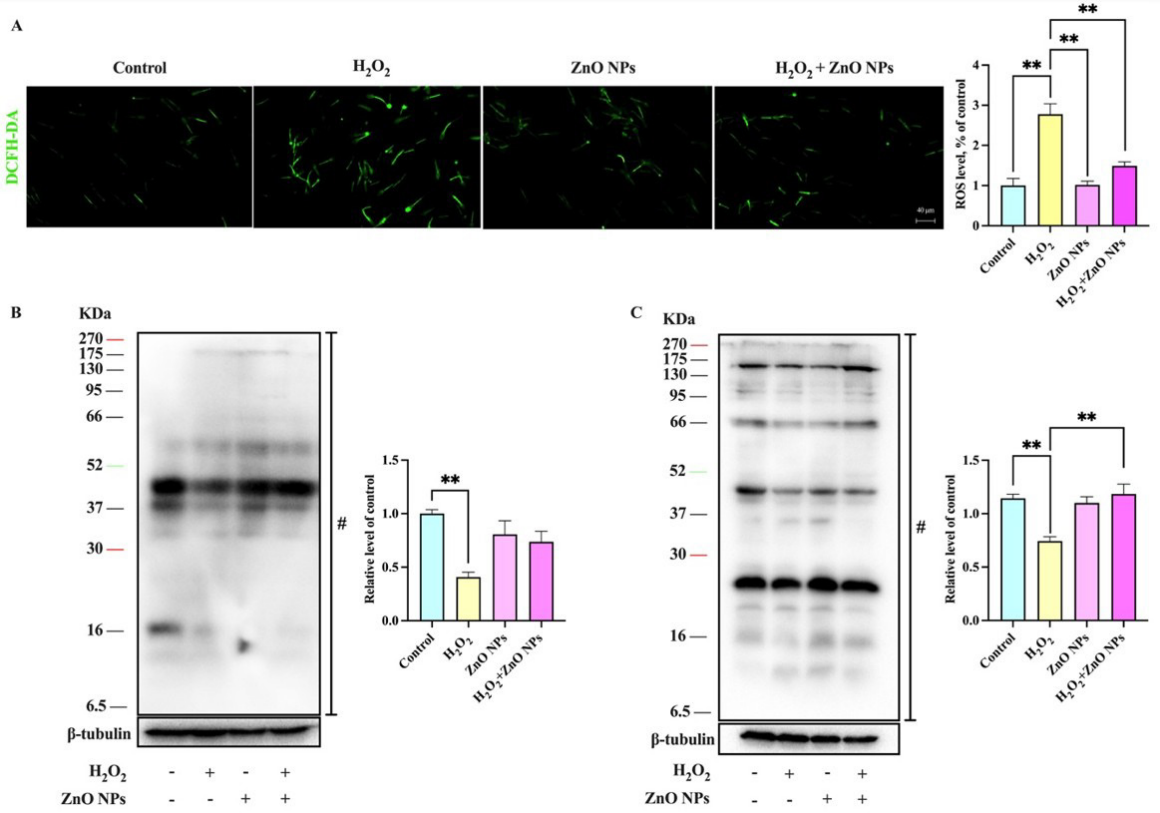
ZnO NPs could mitigate H_2_O_2_-induced P-PKAs decreased. (A) supplement of 100 μM H_2_O_2_ to the basal extender and incubated at 37°C for 2h could induce the accumulation of ROS in boar sperm, and 1 μg/mL ZnO NPs-treated could abate the ROS content. (B) Western blot analysis was performed using an anti-phosphotyrosine antibody. (C) Western blot analysis was performed using an anti-phospho-PKA substrate antibody. The bands used for histogram quantification are labeled #. β-Tubulin was used as an internal control. The experiment was performed at least three times; the presented image is representative and repeatable. (n = 3, ** *P* < 0.01).

To gain insight into how ZnO NPs interacts with the boar sperm, we used the oxidant H_2_O_2_ to obtain a predicted accumulation of ROS in boar sperm, which revealed 100 μM H_2_O_2_ could through ROS inhibit protein phosphorylation (Figure 8). When 1μg/mL ZnO NPs was added to the basal extender in the presence of 100 μM H_2_O_2_, the level of P-PKAs was noticeably higher (*P* < 0.01), but PTP has no significant difference than in the control. It is shown that ZnO NPs could improve sperm quality by suppressing ROS-induced P-PKAs depletion.

## Discussion

### ZnO NPs could improve the quality of boar sperm

As a crucial component of AI programs, liquid storage of sperm is one of the effective methods for the storage of genetic resources and makes a substantial contribution to the advancement of animal husbandry and medical research. Liquid storage includes low-temperature (4°C) and room-temperature storage (17°C) (Ros-Santaella & Pintus, 2021), the corresponding extender is different. In actuality, maintaining a constant temperature in the storage environment is tough. Temperature control is easier for room-temperature storage, but the storage period is shorter than for low-temperature storage. Finding a basal extender suitable for both 4°C and 17°C is necessary. In our experiments, each ejaculate was diluted in a basal medium containing 7 compounds (Table 1). After 7 days of liquid storage at 4°C and 17°C, sperm viability, and motility were all above 60%, and the abnormality rate was under 20%. Thus, we speculated that the basal extender could be suitable for 4°C and 17°C. This extender has a longer storage duration than conventional room-temperature extenders and is far more practical to use in daily life than other low-temperature extenders because it does not require the addition of additional antifreeze ingredients like egg yolk and glycerin.

Liquid storage prolongs sperm lifetime and facilitates greater utilization of sperm, but the quality of preserved sperm decreased (Figure 1). The quality of preserved sperm may be influenced by a number of variables, such as the species, ejaculates, extenders, and preservation techniques (Pezo et al., 2019). According to earlier research, excessive ROS accumulation and OS pose serious threats to the quality of sperm (Drevet & Aitken, 2020; Kim et al., 2011; Wang et al., 2018). ROS levels can be controlled by taking antioxidants that scavenge ROS. The past decade has witnessed enormous research efforts undertaken in the development of nanosized particulate platforms for biological studies (Zhang et al., 2015). ZnO NPs is a acknowledged antioxidant with strong antioxidant properties that can stop tissue dysfunction brought on by OS.

**Figure 9:**
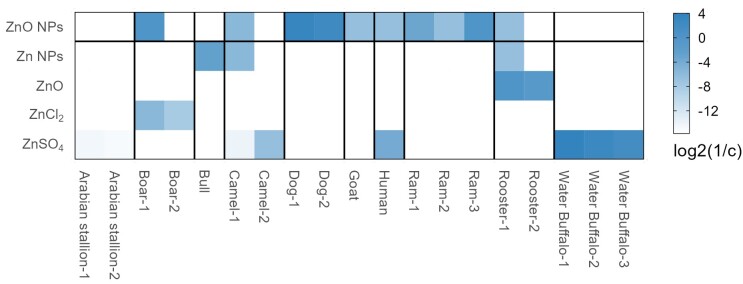
Integrated network analysis to explore the suitable zinc compounds in sperm storage.

In the present study, different concentrations of ZnO NPs were added to the basal extenders in order to investigate whether ZnO NPs could improve the quality of liquid-storage boar sperm. The results showed that after 7 days of liquid storage, the quality of sperm was improved by the addition of 1 μg/mL ZnO NPs. Research demonstrated that both particle and ionized ZnO NPs can be taken up by cells (Jeon et al., 2020). The nano-sized preparation potentiates the effects of zinc compared to the regular-size particles of zinc compounds (Barkhordari et al., 2013; Dorostkar et al., 2014; Zhandi et al., 2020). The cluster analysis of ZnO NPs compared with other types of zinc compounds revealed that the nanomaterial had a greater bioavailability in sperm preservation (Abedin et al., 2023; Arangasamy et al., 2018; Dorostkar et al., 2014; Fayez et al., 2023; Ghallab et al., 2017; Heidari et al., 2019; Hu, 2023; Isaac et al., 2017; Jahanbin et al., 2021; Khodaei-Motlagh et al., 2022; Kotdawala et al., 2012; Li et al., 2023; Marini et al., 2023; Shahin et al., 2020; Soltani et al., 2022; Zhandi et al., 2020) (Figure 9). As most functions of sperm are closely related to the sperm plasma membrane, ZnO NPs may improve sperm quality by creating a favorable environment with the higher PMI. Zinc may interact with some functional groups of the essential component of the sperm membrane (Sørensen et al., 1999), which may explain the higher PMI after storage with 1 μg/mL ZnO NPs in the extender. Sperm motility and viability are linked to PMI, which is influenced by zinc concentration (Kumar et al., 2006). The addition of 10 μg/mL ZnO NPs increased the amount of morphologically abnormal sperm at 17°C in comparison to the control group (*P* < 0.05) (Figure 3J), suggesting that higher concentrations of ZnO NPs may be harmful to sperm morphology. Given these findings, our results indicated that ZnO NPs could be used as one of the exogenous additives for maintaining the fertility of boar semen for liquid storage, this is consistent with previous studies of zinc (Barkhordari et al., 2013; Choi & Choy, 2014; Dorostkar et al., 2014; Isaac et al., 2017).

### ZnO NPs could improve the the antioxidant capabilities of boar sperm

As a by-product of mitochondrial oxidative phosphorylation, ROS causes oxidative damage in mitochondria during liquid storage (Piomboni et al., 2012). The low level of ROS is necessary for the physiological function of sperm including the capacitation, hyperactivation, and acrosomal reaction (Barati et al., 2020; Herrero et al., 2003). Nevertheless, the accumulation of ROS may cause sperm membrane lipid peroxidation, resulting in a drop in sperm PMI and MMP and an increase in MDA (the membranous peroxidation marker) levels during liquid storage. The antioxidant enzyme system of sperm consists of the SOD, GSH-Px, and CAT (Shiva et al., 2011). Zinc as an antioxidant by catalyzing the action of copper/zinc SOD, stabilizing cytoplasmic membrane structure, protecting protein sulfhydryl groups, and increasing the expression of metallothionein, which has a metal-binding capability as well as antioxidant capabilities (Lee, 2018; Olechnowicz et al., 2018). The findings indicated that ZnO NPs might boost the activity of antioxidant enzymes such as SOD, GSH-Px, and CAT (Gharaei et al., 2020; Liu et al., 2022), and adding zinc to the testicular cell culture increases the enzymatic activity of Cu/Zn SOD (Celino et al., 2011), which were consistent with the effects of ZnO NPs on sperm liquid preservation in our study. In the present study, the addition of 1 μg/mL ZnO NPs increased the activities of these enzymes and enhanced the antioxidant capacity of sperm, resulting in an increase in MMP and a decrease in MDA content. The protection of ZnO NPs on the sperm plasma membrane (Figure 3C, H) helps to mitigate the detrimental impact on critical organelles like mitochondria. The improved sperm quality observed in study may be attributed to ZnO NPs increased sperm’s antioxidant capacity. Furthermore, lower MDA level could be due to zinc antioxidant capacity reducing ROS released from sperm mitochondria or preventing the accumulation of LPO products during preservation. Taken together, we logically concluded that the protection provided by ZnO NPs to boar sperm during liquid storage may be due to the antioxidant properties of ZnO NPs.

### ZnO NPs could protect sperm from OS by inhibiting protein dephosphorylation

Research indicates that the P-PKAs and PTP levels in sperm positively correlated with sperm motility (Fu et al., 2018; Li et al., 2019). However, whether ZnO NPs impacts sperm protein phosphorylation modifications is still not completely understood. In the present study, sperm P-PKAs level decreased significantly after 7 days of liquid storage, but PTP level were not significantly different compared to fresh sperm. Increased PTP level is one of the hallmarks of sperm capacitation, a terminal event in which only a small number of capacitated sperm combine with the egg to create a zygote, and the majority of sperm perish after capacitation (Signorelli et al., 2012; Sutovsky et al., 2019). As a result, sperm was not pre-capacitated during liquid storage, that is, before entering the female reproductive system, which was advantageous in extending the liquid storage period. Interestingly, because zinc signature is an early signal of sperm capacitation and a possible biomarker of sperm quality/fertility (Kerns et al., 2018), ZnO NPs supplements may prevent sperm from becoming pre-capacitated.

**Figure 10:**
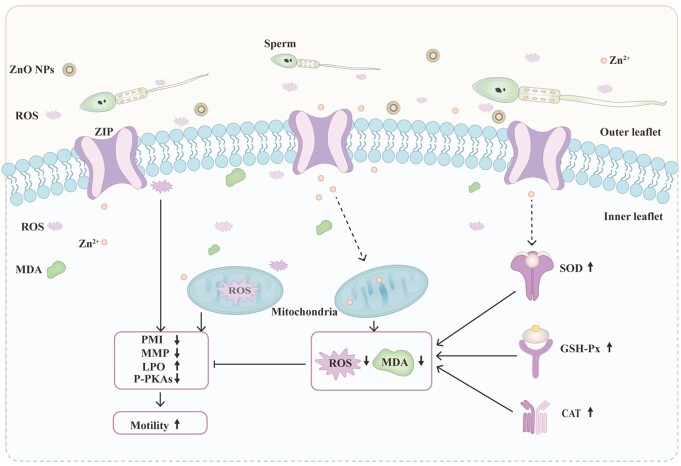
Putative mechanisms by which ZnO NPs protects liquid storage boar sperm from ROS-induced functional damage. ZnO NPs may release zinc ions to the basal extender, and zinc ions enter the sperm through zinc influx transporters (ZIP) on the sperm membrane to reduce intracellular ROS levels. Decreasing intracellular ROS levels may enhance the MMP and P-PKAs levels and enhance cellular ATP levels, subsequently increasing the motility of spermatozoa in liquid storage.

After 7 days of liquid storage, the level of P-PKAs was noticeably higher in the treatment groups than in the control group. Moreover, the trend was more noticeable after 7 days at 4°C than at 17°C, demonstrating that the change in P-PKAs level in boar sperm between 52 ~ 95 kd was related to storage temperature (Figure 7B). It is noteworthy that sperm rely on post-translational modifications (PTM) more than any other cell type because that mature sperm are transcriptionally and translationally silent (Baker, 2016; Mostek-Majewska et al., 2023; Pitnick et al., 2020). Sperm protein phosphorylation is one of the most important PTM of sperm, which seems to play a much greater role, leading, depending on the levels of ROS. Moreover, suitable ROS levels are essential for the level of protein phosphorylation. Low levels of ROS induce protein phosphorylation (Aitken et al., 1998). In contrast, high levels of ROS inhibit the synthesis of adenosine cyclase and promote protein dephosphorylation (Dimitriadis et al., 2008). In order to further explore whether there was a relationship between ROS accumulation and the protein phosphorylation affected by ZnO NPs, we used H_2_O_2_ to generate excessive amounts of ROS in sperm. When 100 μM H_2_O_2_ was added to the basal extender, the overproduction of ROS accelerated protein dephosphorylation (Li et al., 2019), resulting in P-PKAs and PTP levels that were much lower than in the control group. Moreover, the results showed that ZnO NPs could alleviate the decrease in P-PKAs caused by H_2_O_2_ (Figure 8A). Thus, it is logical to conclude that ZnO NPs could protect sperm from OS by inhibiting protein dephosphorylation induced by ROS or/and by scavenging excess ROS (Figure 10).

## Conclusion

In conclusion, this study demonstrated that ZnO NPs represents a promising compound for the reduction of ROS in extenders for boar semen preservation at 4°C and 17°C for the first time, resulting in a possible improvement in the quality of liquid-storage boar sperm. In particular, PKAs dephosphorylation, a molecular mechanism, contributed to the protective effect of ZnO NPs against ROS toxicity. The current study provides a novel substitute for the liquid storage techniques for boar sperm, contributing to promoting the application of ZnO NPs in breeding livestock in the future. Furthermore, more research is needed to evaluate whether ZnO NPs supplementation influences fertilization and embryo development.

## References

[B001] Abdelbaky AS, Abd El-Mageed TA, Babalghith AO, Selim S, Mohamed A (2022). Green synthesis and characterization of ZnO nanoparticles using pelargonium odoratissimum (L.) aqueous leaf extract and their antioxidant, antibacterial and anti-inflammatory activities. Antioxidants.

[B002] Abedin SN, Baruah A, Baruah KK, Kadirvel G, Katiyar R, Khargharia G, Bora A, Dutta DJ, Sinha S, Tamuly S, Phookan A, Deori S (2023). In vitro and in vivo studies on the efficacy of zinc-oxide and selenium nanoparticle in cryopreserved goat (Capra hircus) spermatozoa. Biol Trace Elem Res.

[B003] Aitken RJ, Clarkson JS, Fishel S (1989). Generation of reactive oxygen species, lipid peroxidation, and human sperm function. Biol Reprod.

[B004] Aitken RJ, Harkiss D, Knox W, Paterson M, Irvine DS (1998). A novel signal transduction cascade in capacitating human spermatozoa characterised by a redox-regulated, cAMP-mediated induction of tyrosine phosphorylation. J Cell Sci.

[B005] Arangasamy A, Krishnaiah MV, Manohar N, Selvaraju S, Rani GP, Soren NM, Reddy IJ, Ravindra JP (2018). Cryoprotective role of organic Zn and Cu supplementation in goats (Capra hircus) diet. Cryobiology.

[B006] Azarin K, Usatov A, Minkina T, Plotnikov A, Kasyanova A, Fedorenko A, Duplii N, Vechkanov E, Rajput VD, Mandzhieva S, Alamri S (2022). Effects of ZnO nanoparticles and its bulk form on growth, antioxidant defense system and expression of oxidative stress related genes in Hordeum vulgare L. Chemosphere.

[B007] Baker MA (2016). Proteomics of post-translational modifications of mammalian spermatozoa. Cell Tissue Res.

[B008] Barati E, Nikzad H, Karimian M (2020). Oxidative stress and male infertility: current knowledge of pathophysiology and role of antioxidant therapy in disease management. Cell Mol Life Sci.

[B009] Barkhordari A, Hekmatimoghaddam S, Jebali A, Khalili MA, Talebi A, Noorani M (2013). Effect of zinc oxide nanoparticles on viability of human spermatozoa. Iran J Reprod Med.

[B010] Bashandy SAE, Alaamer A, Moussa SAA, Omara EA (2018). Role of zinc oxide nanoparticles in alleviating hepatic fibrosis and nephrotoxicity induced by thioacetamide in rats. Can J Physiol Pharmacol.

[B011] Celino FT, Yamaguchi S, Miura C, Ohta T, Tozawa Y, Iwai T, Miura T (2011). Tolerance of spermatogonia to oxidative stress is due to high levels of Zn and Cu/Zn superoxide dismutase. PLoS One.

[B012] Choi SJ, Choy JH (2014). Biokinetics of zinc oxide nanoparticles: toxicokinetics, biological fates, and protein interaction. Int J Nanomedicine.

[B013] Didion BA, Braun GD, Duggan MV (2013). Field fertility of frozen boar semen: a retrospective report comprising over 2600 AI services spanning a four year period. Anim Reprod Sci.

[B014] Dimitriadis F, Giannakis D, Pardalidis N, Zikopoulos K, Paraskevaidis E, Giotitsas N, Kalaboki V, Tsounapi P, Baltogiannis D, Georgiou I, Saito M, Watanabe T, Miyagawa I, Sofikitis N (2008). Effects of phosphodiesterase-5 inhibitors on sperm parameters and fertilizing capacity. Asian J Androl.

[B015] Dorostkar K, Alavi Shoushtari SM, Khaki A (2014). Effects of In vitro zinc sulphate additive to the semen extender on water buffalo (Bubalusbubalis) spermatozoa before and after freezing. Int J Fertil Steril.

[B016] Drevet JR, Aitken RJ (2020). Oxidation of sperm nucleus in mammals: a physiological necessity to some extent with adverse impacts on oocyte and offspring. Antioxidants.

[B017] Erfani Majd N, Tabandeh MR, Hosseinifar S, Rahimi Zarneh S (2021). Chemical and green ZnO nanoparticles ameliorated adverse effects of cisplatin on histological structure, antioxidant defense system and neurotrophins expression in rat hippocampus. J Chem Neuroanat.

[B018] Faizan M, Bhat JA, Chen C, Alyemeni MN, Wijaya L, Ahmad P, Yu F (2021). Zinc oxide nanoparticles (ZnO-NPs) induce salt tolerance by improving the antioxidant system and photosynthetic machinery in tomato. Plant Physiol Biochem.

[B019] Farhana M, Munis MFH, Alamer KH, Althobaiti AT, Kamal A, Liaquat F, Haroon U, Ahmed J, Chaudhary HJ, Attia H (2022). ZnO nanoparticle-mediated seed priming induces biochemical and antioxidant changes in chickpea to alleviate fusarium wilt. J Fungi (Basel).

[B020] Fayez E, El Sayed M, Rawash ZM, Salama A (2023). Influence of the addition of zinc oxide nanoparticles to cryopreservation medium for dog epididymal spermatozoa. Top Companion Anim Med.

[B021] Fu J, Yang Q, Li Y, Li P, Wang L, Li X (2018). A mechanism by which Astragalus polysaccharide protects against ROS toxicity through inhibiting the protein dephosphorylation of boar sperm preserved at 4 °C. J Cell Physiol.

[B022] Gadani B, Bucci D, Spinaci M, Tamanini C, Galeati G (2017). Resveratrol and Epigallocatechin-3-gallate addition to thawed boar sperm improves in vitro fertilization. Theriogenology.

[B023] Ghallab AM, Shahat AM, Fadl AM, Ayoub MM, Moawad AR (2017). Impact of supplementation of semen extender with antioxidants on the quality of chilled or cryopreserved Arabian stallion spermatozoa. Cryobiology.

[B024] Gharaei A, Khajeh M, Khosravanizadeh A, Mirdar J, Fadai R (2020). Fluctuation of biochemical, immunological, and antioxidant biomarkers in the blood of beluga (Huso huso) under effect of dietary ZnO and chitosan-ZnO NPs. Fish Physiol Biochem.

[B025] Heidari J, Seifdavati J, Mohebodini H, Sharifi RS, Benemar HA (2019). Effect of nano zinc oxide on post-thaw variables and oxidative status of moghani ram semen. Kafkas Univ Vet Fak Derg.

[B026] Herrero MB, de Lamirande E, Gagnon C (2003). Nitric oxide is a signaling molecule in spermatozoa. Curr Pharm Des.

[B027] Hu Q (2023). Effects of zinc chloride on boar sperm quality during liquid storage at 17°C. Vet Med Sci.

[B028] Isaac AV, Kumari S, Nair R, Urs DR, Salian SR, Kalthur G, Adiga SK, Manikkath J, Mutalik S, Sachdev D, Pasricha R (2017). Supplementing zinc oxide nanoparticles to cryopreservation medium minimizes the freeze-thaw-induced damage to spermatozoa. Biochem Biophys Res Commun.

[B029] Jahanbin R, Yazdanshenas P, Rahimi M, Hajarizadeh A, Tvrda E, Nazari SA, Mohammadi-Sangcheshmeh A, Ghanem N (2021). In vivo and in vitro evaluation of bull semen processed with Zinc (Zn) nanoparticles. Biol Trace Elem Res.

[B030] Jeon YR, Yu J, Choi SJ (2020). Fate determination of ZnO in commercial foods and human intestinal cells. Int J Mol Sci.

[B031] Jiang J, Pi J, Cai J (2018). The advancing of zinc oxide nanoparticles for biomedical applications. Bioinorg Chem Appl.

[B032] Kalaimurugan D, Lalitha K, Durairaj K, Sivasankar P, Park S, Nithya K, Shivakumar MS, Liu WC, Balamuralikrishnan B, Venkatesan S (2022). Biogenic synthesis of ZnO nanoparticles mediated from Borassus flabellifer (Linn): antioxidant, antimicrobial activity against clinical pathogens, and photocatalytic degradation activity with molecular modeling. Environ Sci Pollut Res Int.

[B033] Kerns K, Zigo M, Drobnis EZ, Sutovsky M, Sutovsky P (2018). Zinc ion flux during mammalian sperm capacitation. Nat Commun.

[B034] Khodaei-Motlagh M, Masoudi R, Karimi-Sabet MJ, Hatefi A (2022). Supplementation of sperm cooling medium with Zinc and Zinc oxide nanoparticles preserves rooster sperm quality and fertility potential. Theriogenology.

[B035] Kim S, Lee YJ, Kim YJ (2011). Changes in sperm membrane and ROS following cryopreservation of liquid boar semen stored at 15 °C. Anim Reprod Sci.

[B036] Kotdawala AP, Kumar S, Salian SR, Thankachan P, Govindraj K, Kumar P, Kalthur G, Adiga SK (2012). Addition of zinc to human ejaculate prior to cryopreservation prevents freeze-thaw-induced DNA damage and preserves sperm function. J Assist Reprod Genet.

[B037] Kumar N, Verma RP, Singh LP, Varshney VP, Dass RS (2006). Effect of different levels and sources of zinc supplementation on quantitative and qualitative semen attributes and serum testosterone level in crossbred cattle (Bos indicus x Bos taurus) bulls. Reprod Nutr Dev.

[B038] Lee SR (2018). Critical role of zinc as either an antioxidant or a prooxidant in cellular systems. Oxid Med Cell Longev.

[B039] Li R, Wu X, Zhu Z, Lv Y, Zheng Y, Lu H, Zhou K, Wu D, Zeng W, Dong W, Zhang T (2022). Polyamines protect boar sperm from oxidative stress in vitro. J Anim Sci.

[B040] Li RN, Zhu ZD, Zheng Y, Lv YH, Tian XE, Wu D, Wang YJ, Zeng WX (2020). Metformin improves boar sperm quality via 5′-AMP-activated protein kinase-mediated energy metabolism in vitro. Zool Res.

[B041] Li X, Wang L, Liu H, Fu J, Zhen L, Li Y, Zhang Y, Zhang Y (2019). C(60) fullerenes suppress reactive oxygen species toxicity damage in boar sperm. Nano-Micro Lett.

[B042] Li Y, Qin S, Cui W, Zhao F, He M, Jiang Z (2023). Progress on the roles of zinc in sperm cryopreservation. Theriogenology.

[B043] Liu ZM, Faizan M, Chen C, Zheng LH, Yu FY (2022). The combined analysis of transcriptome and antioxidant enzymes revealed the mechanism of EBL and ZnO NPs enhancing styrax tonkinensis seed abiotic stress resistance. Genes (Basel).

[B044] Mahesh S, Narasaiah BP, Himabindu B, Balaji GL, Pradeepkiran JA, Padhy H (2022). Sunflower-assisted bio-derived ZnO-NPs as an efficient nanocatalyst for the synthesis of novel quinazolines with highly antioxidant activities. Antioxidants.

[B045] Marini P, Fernández Beato L, Cane F, Teijeiro JM (2023). Effect of zinc on boar sperm liquid storage. Front Vet Sci.

[B046] Mostek-Majewska A, Majewska A, Janta A, Ciereszko A (2023). New insights into posttranslational modifications of proteins during bull sperm capacitation. Cell Commun Signal.

[B047] Olechnowicz J, Tinkov A, Skalny A, Suliburska J (2018). Zinc status is associated with inflammation, oxidative stress, lipid, and glucose metabolism. J Physiol Sci.

[B048] Pezo F, Romero F, Zambrano F, Sánchez RS (2019). Preservation of boar semen: an update. Reprod Domest Anim.

[B049] Piomboni P, Focarelli R, Stendardi A, Ferramosca A, Zara V (2012). The role of mitochondria in energy production for human sperm motility. Int J Androl.

[B050] Pitnick S, Wolfner MF, Dorus S (2020). Post-ejaculatory modifications to sperm (PEMS). Biol Rev Camb Philos Soc.

[B051] Ren F, Fang Q, Feng TY, Li Y, Wang YH, Zhu HJ, Hu JH (2019). Lycium barbarum and Laminaria japonica polysaccharides improve Cashmere goat sperm quality and fertility rate after cryopreservation. Theriogenology.

[B052] Ros-Santaella JL, Pintus E (2021). Plant extracts as alternative additives for sperm preservation. Antioxidants.

[B053] Shahin MA, Khalil WA, Saadeldin IM, Swelum AA, El-Harairy MA (2020). Comparison between the effects of adding vitamins, trace elements, and nanoparticles to SHOTOR extender on the cryopreservation of dromedary camel epididymal spermatozoa. Animals (Basel).

[B054] Shiva M, Gautam AK, Verma Y, Shivgotra V, Doshi H, Kumar S (2011). Association between sperm quality, oxidative stress, and seminal antioxidant activity. Clin Biochem.

[B055] Signorelli J, Diaz ES, Morales P (2012). Kinases, phosphatases and proteases during sperm capacitation. Cell Tissue Res.

[B056] Soltani L, Samereh S, Mohammadi T (2022). Effects of different concentrations of zinc oxide nanoparticles on the quality of ram cauda epididymal spermatozoa during storage at 4°C. Reprod Domest Anim.

[B057] Sørensen MB, Bergdahl IA, Hjøllund NH, Bonde JP, Stoltenberg M, Ernst E (1999). Zinc, magnesium and calcium in human seminal fluid: relations to other semen parameters and fertility. Mol Hum Reprod.

[B058] Sutovsky P, Kerns K, Zigo M, Zuidema D (2019). Boar semen improvement through sperm capacitation management, with emphasis on zinc ion homeostasis. Theriogenology.

[B059] Wang S, Sun M, Wang N, Yang K, Guo H, Wang J, Zhang Y, Yue S, Zhou J (2018). Effects of L-glutamine on boar sperm quality during liquid storage at 17°C. Anim Reprod Sci.

[B060] Watson PF (2000). The causes of reduced fertility with cryopreserved semen. Anim Reprod Sci.

[B061] White IG (1993). Lipids and calcium uptake of sperm in relation to cold shock and preservation: a review. Reprod Fertil Dev.

[B062] Yohan D, Chithrani BD (2014). Applications of nanoparticles in nanomedicine. J Biomed Nanotechnol.

[B063] Zhandi M, Talebnia-Chalanbar A, Towhidi A, Sharafi M, Yousefi AR, Hussaini SMH (2020). The effect of zinc oxide on rooster semen cryopreservation. Br Poult Sci.

[B064] Zhang S, Gao H, Bao G (2015). Physical principles of nanoparticle cellular endocytosis. ACS Nano.

[B065] Zhang X, Guo SM, Zhu DW, Li Y, Wen F, Xian M, Hu ZT, Zou QL, Zhang LK, Chen YL, Hu JH (2023). Metformin improves sheep sperm cryopreservation via vitalizing the AMPK pathway. Theriogenology.

[B066] Zhu Z, Kawai T, Umehara T, Hoque SAM, Zeng W, Shimada M (2019). Negative effects of ROS generated during linear sperm motility on gene expression and ATP generation in boar sperm mitochondria. Free Radic Biol Med.

[B067] Zhu Z, Li R, Fan X, Lv Y, Zheng Y, Hoque SAM, Wu D, Zeng W (2019). Resveratrol improves boar sperm quality via 5'AMP-Activated protein kinase activation during cryopreservation. Oxid Med Cell Longev.

[B068] Zhu ZD, Li RN, Wang LQ, Zheng Y, Hoque A, Lv YH, Zeng WX (2019). Glycogen synthase Kinase-3 regulates sperm motility and acrosome reaction via affecting energy metabolism in goats. Front Physiol.

